# Impact of the Madden-Julian oscillation on Antarctic sea ice and its dynamical mechanism

**DOI:** 10.1038/s41598-019-47150-3

**Published:** 2019-07-24

**Authors:** Hyun-Ju Lee, Kyong-Hwan Seo

**Affiliations:** 1 Department of Atmospheric Sciences, Division of Earth Environmental Systems, Pusan National University, Pusan, Korea; 2 Research Center for Climate Sciences, Pusan National University, Pusan, Korea

**Keywords:** Climate-change impacts, Atmospheric dynamics

## Abstract

The influence of the Madden-Julian oscillation (MJO) on Antarctic sea ice extent has not been extensively explored. This study investigates intraseasonal variability of the sea ice extent induced by the MJO and its physical mechanism. During austral winter, the sea ice extent anomaly exhibits considerable melting and freezing as the MJO evolves. Numerical experiments and the Rossby wave theory show that the high-latitude circulation anomalies in response to the MJO are responsible for the sea ice change. The MJO-induced Rossby waves propagate into the Southern Hemisphere through the northerly ducts over the western Indian Ocean–central Africa and the Maritime Continent. The MJO-induced circulation anomalies reach high latitudes and lead to anomalous meridional temperature advection, causing changes in the sea ice extent. The time difference between the meridional wind and sea ice anomalies is ~5 days. As the MJO moves, the sea ice extent anomaly also exhibits eastward-migrating behavior. Strong sea ice melting in the total anomaly is synchronous to the evolution of the MJO, suggesting the practical usefulness of the location of the MJO for the prediction of the sea ice decrease.

## Introduction

Sea ice in the polar region plays a crucial role in modulating the stratospheric polar vortex^1,2^, the Earth surface albedo^3^, the thermal interaction between atmosphere and ocean^4,5^, and the local ecosystems^6–8^. Antarctic sea ice is found to be affected by atmospheric and oceanic internal modes on the seasonal to interannual and interdecadal time scales, such as the El Niño–Southern Oscillation (ENSO)^9^, Southern Annular Mode^10,11^, Antarctic Circumpolar Wave^12^, and Atlantic Multidecadal Oscillation^13^. These variabilities induce high-latitude circulation anomalies, leading to warm or cold air advection, anomalous sea ice motion, and ocean current change, and ultimately changes in the sea ice. On the intraseasonal time scale, there may be atmospheric or oceanic processes responsible for the changes in Antarctic sea ice. In particular, remote forcing from the tropics to the polar region needs to be examined.

This study demonstrates the impact of the Madden–Julian oscillation (MJO) on the intraseasonal variability of Antarctic sea ice. The MJO is the dominant physical mode on the intraseasonal time scale with planetary-scale circulation coupled with a large-scale convective complex in the tropics^14^. The MJO is known as a source of the subseasonal-to-seasonal predictability of weather and climate^15^. Many previous studies have explored the effects of the MJO on global circulation^16,17^, regional precipitation^18,19^, and extratropical temperature^20–23^. Recently, the MJO is proposed to influence the polar region^24–28^. For example, a study showed that enhanced diabatic heating over the far western Pacific induces warming over the Arctic through the zonal mean fluid dynamics^26^, and therefore, it is anticipated that the sea ice over this region can be changed^29^. There were also earlier studies to examine the MJO effect on the change in Antarctic sea ice^30,31^. However, these were on a short intraseasonal time scale (i.e., a period of 10–15 days), and its impact was not found. The latter arises because an MJO index that is not separated by season was used, thus, the genuine seasonal effect was not captured well. While the MJO moves eastward along the equator during boreal winter, northward or northwestward propagation occurs along with the typical eastward movement during austral winter^32^. Additionally, it is likely that the synoptic-scale internal variability in the extratropics is larger than the atmospheric response forced from the tropics on this rather short intraseasonal time scale^31^. In this study, we particularly investigate the effects of the tropical lower frequency variation with time scale ranging from 20 to 90 days. The time scale has a significant impact on the extratropical circulation and teleconnection, and, in fact, it is commonly used in studies associated with the MJO-related teleconnection to higher latitudes.

Atmospheric circulation modulates the sea ice concentration and cover^9,10^ immediately or with some time lag through the thermal forcing and the mechanical forcing. The former is related to temperature advection by wind and the latter is related to the effect of wind stress on the sea ice velocity and Ekman drift in the ocean. Between the two, thermal forcing contributes dominantly to the intraseasonal variability of Antarctic sea ice^33^. On the intraseasonal time scale, atmospheric circulation variation correlates coherently with sea ice extent variation^30^. Since the circulation plays an important role, it is essential to investigate the dynamical processes of the MJO-induced circulation response in the Southern Hemisphere.

This study aims to explore the influence of the MJO on Antarctic sea ice extent during austral winter and to investigate the dynamical mechanism generating the large-scale circulation response to the MJO. Since teleconnection pattern due to tropical forcing is prominent in the winter hemisphere due to the existence of strong potential vorticity in the background dynamic field, it is expected that the MJO influences the climate over the Antarctic region. For this, a series of simulations using a linear baroclinic model is performed, and ray tracing for poleward propagating waves is applied based on Rossby wave theory.

## Results

### Characteristics of the MJO and the extratropical circulation response

**Figure 1:**
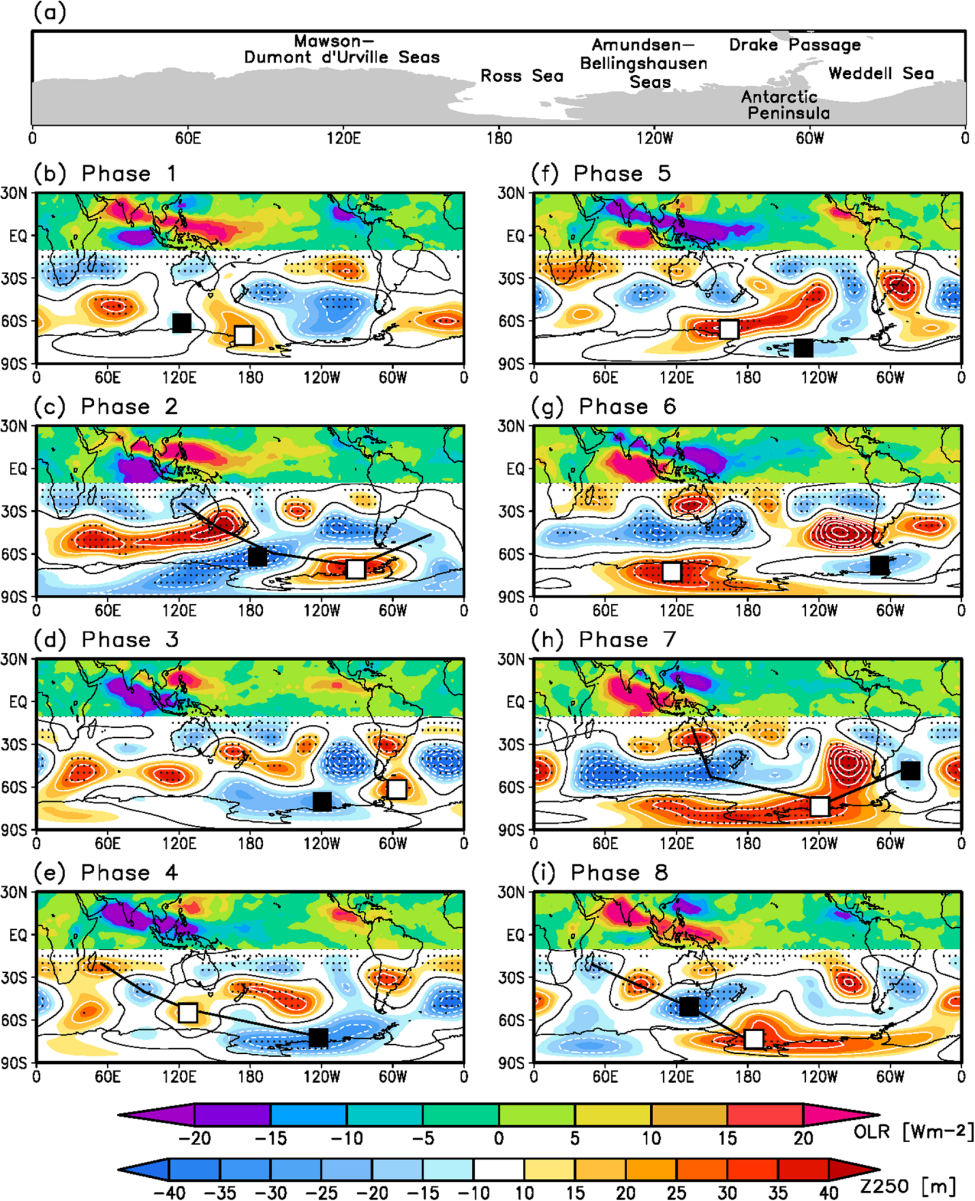
MJO convection and corresponding circulation response for eight MJO phases. (**a**) Geographical location of the Southern Hemisphere polar regions and (**b**–**i**) composite maps of filtered OLR (shading, 10°S to 30°N; W m^−2^) and 250-hPa geopotential height (shading with contour at 0, south of 10°S; m) anomalies. Black (white) squares indicate the centers of the high-latitude cyclonic (anticyclonic) circulation anomalies. Dots represent the statistically significant areas of the geopotential height at the 90% confidence level. Black thick line in (**c**,**e**,**h**,**i**) represents the teleconnection pattern.

To identify the MJO convection and circulation response, a composite analysis of the outgoing longwave radiation (OLR) and 250-hPa geopotential height anomalies is performed with respect to the MJO index (see the Methods section for details about the composite and the definition of the index). During austral winter (June–August, JJA), MJO convection moves northward or northeastward with a northwest-to-southeast-tilted structure. The MJO lifecycle is divided into 8 phases according to their convection location (Fig. 1b–i)^34^. Enhanced (suppressed) convection arises over the Indian Ocean in phase 1 (5) and disappears over the western North Pacific (i.e., South China Sea) in phase 8 (4). When the dipole convection anomalies are located over the Indian Ocean and western North Pacific (e.g., phases 2 and 7), the Pacific-South America (PSA)-like teleconnection pattern develops in the mid-to-high latitudes of the Southern Hemisphere (black solid lines in Fig. 1c,h)^35^. This wave train seems to start from Australia and proceed into the Drake Passage (see the geographical location in Fig. 1a), featuring an anticlockwise propagation pattern. Meanwhile, at phases 4 and 8 at which the convection anomalies exist over the Indian subcontinent and Maritime Continent, a wave train arising from Madagascar and propagating toward the Amundsen-Bellingshausen Seas (black lines in Fig. 1e,i) appears.

Overall, as the MJO advances eastward, the MJO-induced circulation anomaly in the high latitudes (approximately from 80° S to 50° S) also propagates eastward. For example, a cyclonic circulation anomaly (black square in Fig. 1b–i) over the Dumont d’Urville Sea in phase 8 (Fig. 1i) migrates toward the east during the subsequent phases (i.e., phases 1–6, Fig. 1b–g), finally reaching the Weddell Sea in phase 7 (Fig. 1h). Similarly, an anticyclonic circulation anomaly (white square) exhibits behavior analogous to that described above, with different starting phases (i.e., starting over the Dumont d’Urville Sea at phase 4 and migrating eastward afterwards) (Fig. 1e–i,b–d), but more irregular than the cyclonic counterpart. The impact of the circulation response on Antarctic sea ice extent is demonstrated in the next subsection, and the formation mechanism of the circulation anomalies is described in the third subsection.

### The MJO impact on antarctic sea ice

**Figure 2:**
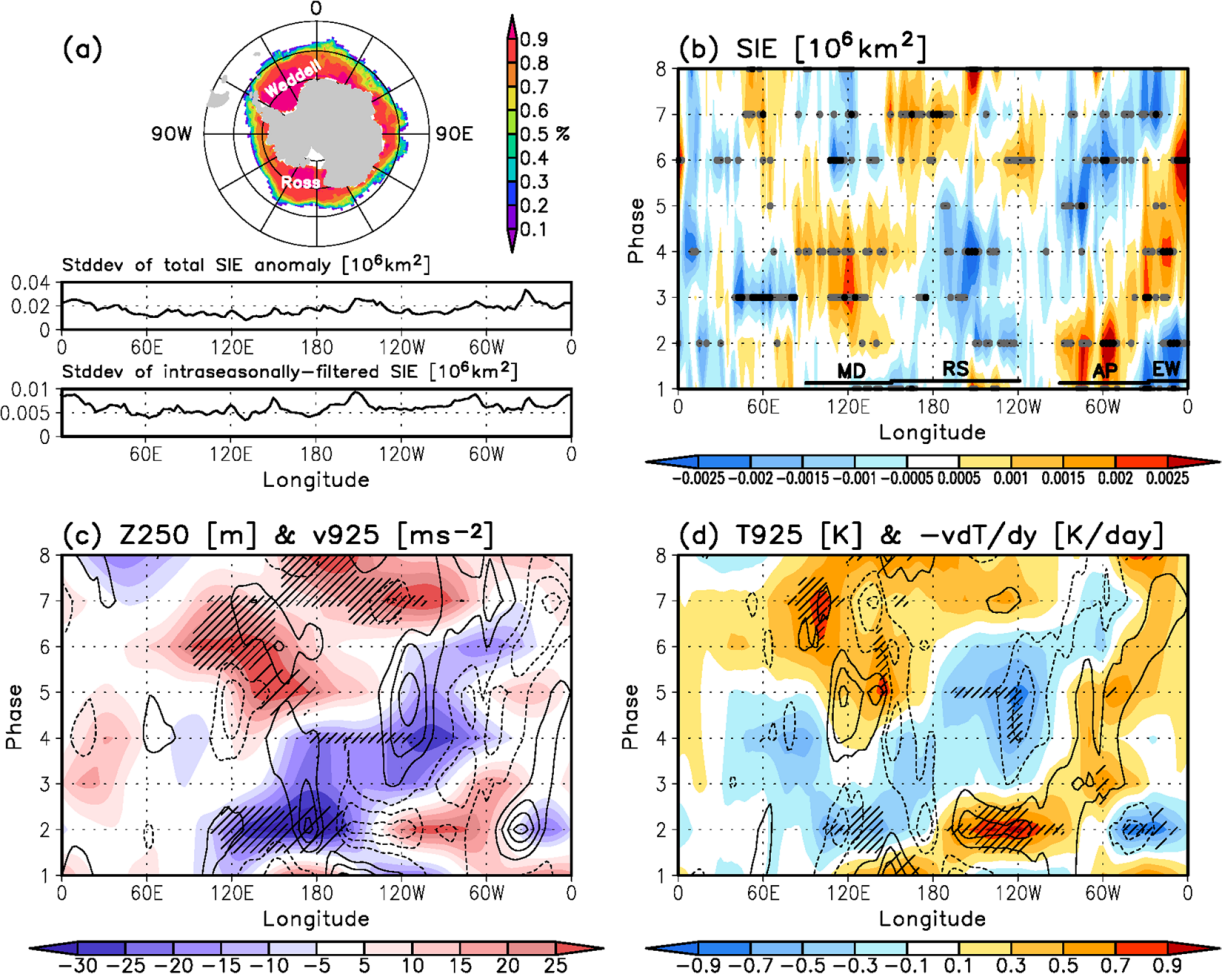
Climatological features of Antarctic sea ice and response of the sea ice and atmospheric variables to the MJO in the Antarctic region. (**a**) (upper) Spatial pattern of JJA-climatological sea ice concentration in the range of 0 to 1 (100%) and standard deviations of (middle) total and (lower) filtered sea ice extent anomalies (×10^6^ km^2^). Composites of filtered (**b**) sea ice extent anomaly (×10^6^ km^2^), (**c**) 250-hPa geopotential height (shading; m) and 925-hPa meridional wind (contour with intervals of 0.3 m s^−1^ and the zero contour is omitted) anomalies, (**d**) 925-hPa air temperature anomaly (shading; K) and the temperature advection by the meridional wind (contour with intervals of 0.3 K day^−1^ and the zero contour is omitted) for eight MJO phases. All atmospheric variables are meridionally averaged from 60° to 80°S. Black and grey dots in (**b**) indicate the significant regions at the 95% and 80% confidence level, respectively. Hatched (densely hatched) areas in (**c**,**d**) represent the significant shading regions at the 90% confidence level, calculated using variables averaged from 60° to 70°S (70° to 80°S, respectively).

Figure 2a shows the spatial pattern of the JJA-mean sea ice concentration (shading in the uppermost plot) and the standard deviations of the total and 20–90-day filtered sea ice extent anomalies (middle and lower plots in Fig. 2a). The austral winter climatological sea ice concentration pattern shows that the areas with the greatest concentration occur over the Ross and Weddell Seas (upper plot in Fig. 2a), where the standard deviations of total sea ice extent anomaly are also large (middle panel in Fig. 2a). The intraseasonal variability of the sea ice extent presents larger standard deviations in West Antarctica than in East Antarctica (lower panel in Fig. 2a). The averaged standard deviation is 0.0066 × 10^6^ km^2^ in West Antarctica and 0.0056 × 10^6^ km^2^ in East Antarctica. The intraseasonal variability accounts for up to 29% of the total variation in the sea ice extent regionally, and on average it explains 20% of the total variation in the Antarctic region (Supplementary Fig. S1).

To investigate the MJO impact on Antarctic sea ice and its physical processes, we plot the composites of the filtered sea ice extent anomaly (Fig. 2b) and those of the atmospheric variables averaged over the high latitudes (Fig. 2c,d) for eight MJO phases. A considerable decrease in the sea ice extent is exhibited in the following regions (see Fig. 2b): 90°E–150°E (Mawson-Dumont d’Urville Seas; MD) in phases 6–8 and 1, 150°E–120°W (Ross Seas; RS) in phases 2–4, 90°W–30°W (sea near the Antarctic Peninsula; AP) in phases 5–7, and 20°W–0°E (eastern Weddell Sea; EW) in phases 1–2. In contrast, a significant increase in the sea ice arises in the same regions at different phases, e.g., MD in phases 3–4, RS in phase 7, AP in phase 2, and EW in phases 3–6. Note that the sea ice extent anomaly displays an eastward propagation as the MJO evolves. This eastward migration is suggestive of the contribution by the eastward moving circulation anomaly generated by the MJO, as seen in Figs 1 and 2c. The circulation response develops anomalous low-level meridional wind (contour in Fig. 2c)—northerly (southerly) to the east (west) of the cyclonic circulation and west (east) of the anticyclonic circulation, inducing the advection of warm and cold air into the Antarctic region (contour in Fig. 2d) and, therefore, the temperature variations (shaded in Fig. 2d).

A temporal difference seems to exist between the sea ice extent and atmospheric variables (Fig. 2b–d). A lead-lag correlation analysis shows that the sea ice extent anomaly correlates significantly with the meridional wind and temperature anomalies with lags of 5 and 4 days, respectively, on Antarctic (with correlation coefficients of 0.55 and −0.37, respectively), although a regional difference exists (see Supplementary Fig. S2). That is, over the high latitudes, the circulation anomaly caused by the MJO forcing induces the temperature anomaly after a day and the sea ice anomaly after 5 days. This is consistent with previous studies^31,36^ that demonstrated that a spatial phase difference appears between the meridional wind and sea ice anomalies, with the former leading the latter by 5 days. The above analysis clearly demonstrates that the atmospheric anomaly drives the sea ice variation.

### The dynamical mechanism generating the circulation anomaly

To demonstrate the dynamic mechanism by which the MJO generates the anomalous circulation in the Antarctic region, we performed a numerical experiment (see Experimental design). Because it takes about two weeks for the circulation response to tropical convection to reach the high latitudes^37^, diabatic forcing preceding two MJO phases is imposed. The simulated upper-tropospheric geopotential height anomaly is plotted by averaging the model simulations of 15–20 days. The results show similar wave train-like anomalies to the observations for all eight MJO phases, although the centers of the circulation anomalies are slightly biased westward (Supplementary Fig. S3). Hereafter, we focus on the response to the forcings of MJO phases 2 and 4, but the same formation mechanism operates in MJO phases 6 and 8 with a sign reversal.

**Figure 3:**
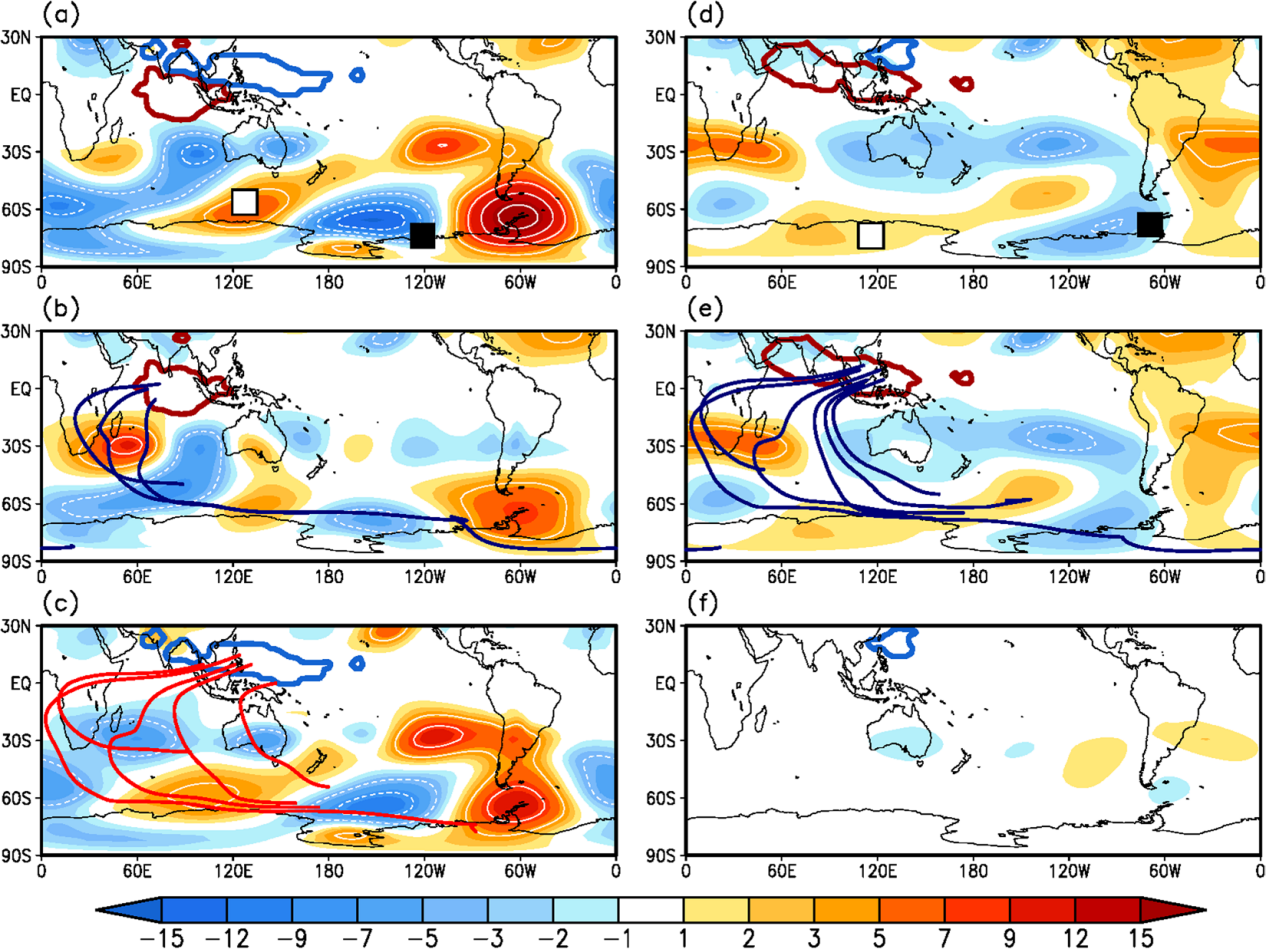
Simulated circulation anomalies in response to the MJO and Rossby wave ray path. Upper-troposphere (σ = 0.229) geopotential height anomaly (shading, zonal mean is subtracted; m) averaged over 15–20 days of model simulation in response to diabatic forcing of MJO (**a**) phase 2, (**d**) phase 4, and its monopole forcing: (**b**,**e**) diabatic heating and (**c**,**f**) diabatic cooling. Simulated circulations correspond to the observed circulation in (left panels) phase 4 and (right panels) phase 6. Black (white) squares in (**a**,**d**) represent centers of the high-latitude cyclonic (anticyclonic) circulation anomalies in the observation, as seen in Fig. 1e,g. Thick red (blue) contours indicate imposed diabatic heating (cooling). In (**b**,**c**,**e**), thick lines represent ray path of the Rossby waves seeded from the (red) diabatic heating and (blue) diabatic cooling regions for initial zonal wavenumber 2.

The result of the model experiment shows that the MJO phase-2 forcing (e.g., enhanced convection over the Indian Ocean and suppressed convection over the western North Pacific) generates a teleconnection pattern, with the anticyclonic circulation anomaly over the MD and the cyclonic circulation anomaly over the RS and Amundsen-Bellingshausen Seas (Fig. 3a). This pattern resembles the circulation anomalies observed at phases 3 and 4 (Fig. 1d,e), although observation (Fig. 1d,e) does not indicate an anticyclonic circulation anomaly over the AP or Drake Passage simulated in the model. Figure 3b,c exhibits the circulation anomaly in response to monopole forcing. Interestingly, the opposite forcing induces the same signed circulation anomalies over nearly the same location, especially at high latitudes (Fig. 3b,c). However, the suppressed convection over the western North Pacific (Fig. 3c) is more responsible for the generation of the total circulation anomaly (Fig. 3a) than the enhanced convection over the Indian Ocean (Fig. 3b). To identify the path of wave energy activity, the ray tracing technique is conducted based on nondivergent barotropic Rossby wave theory (see the Methods section for more details). The waves emitted from the convection over the western North Pacific propagate southward across the equator and reach the Antarctic region (red lines in Fig. 3c). Some waves cross the equator near central Africa, while other waves penetrate via the Maritime Continent. The waves starting from the convection over the Indian Ocean propagate southwestward over central Africa and then travel southeastward (blue lines in Fig. 3b).

On the other hand, the MJO phase-4 forcing (e.g., enhanced convection over the Indian subcontinent/Maritime Continent and weak suppressed convection over the western North Pacific) produces anticyclonic circulation over the MD and cyclonic circulation over the Amundsen-Bellingshausen Seas and AP (Fig. 3d), similar to the circulation anomalies contemporaneously found at phases 5 and 6 (Fig. 1f,g). Separate forcing experiments (Fig. 3e,f) show that enhanced convection (Fig. 3e) produces circulation anomalies similar to the total circulation response (Fig. 3d). Wave rays (blue lines in Fig. 3e) seem to behave as those represented by the red lines in Fig. 3c, as the two are located in similar areas. In fact, however, the waves emanating from the enhanced convection cross the equator near the Maritime continent (not the western Indian Ocean) to reach high latitudes. This suggests that the wave train shifts eastward as the MJO phase proceeds. The suppressed convection is too weak to generate circulation response, as seen in Fig. 3f. Note that antisymmetric circulation and sea ice anomalies develop at MJO phases 6 and 8 with respect to phases 2 and 4.

**Figure 4:**
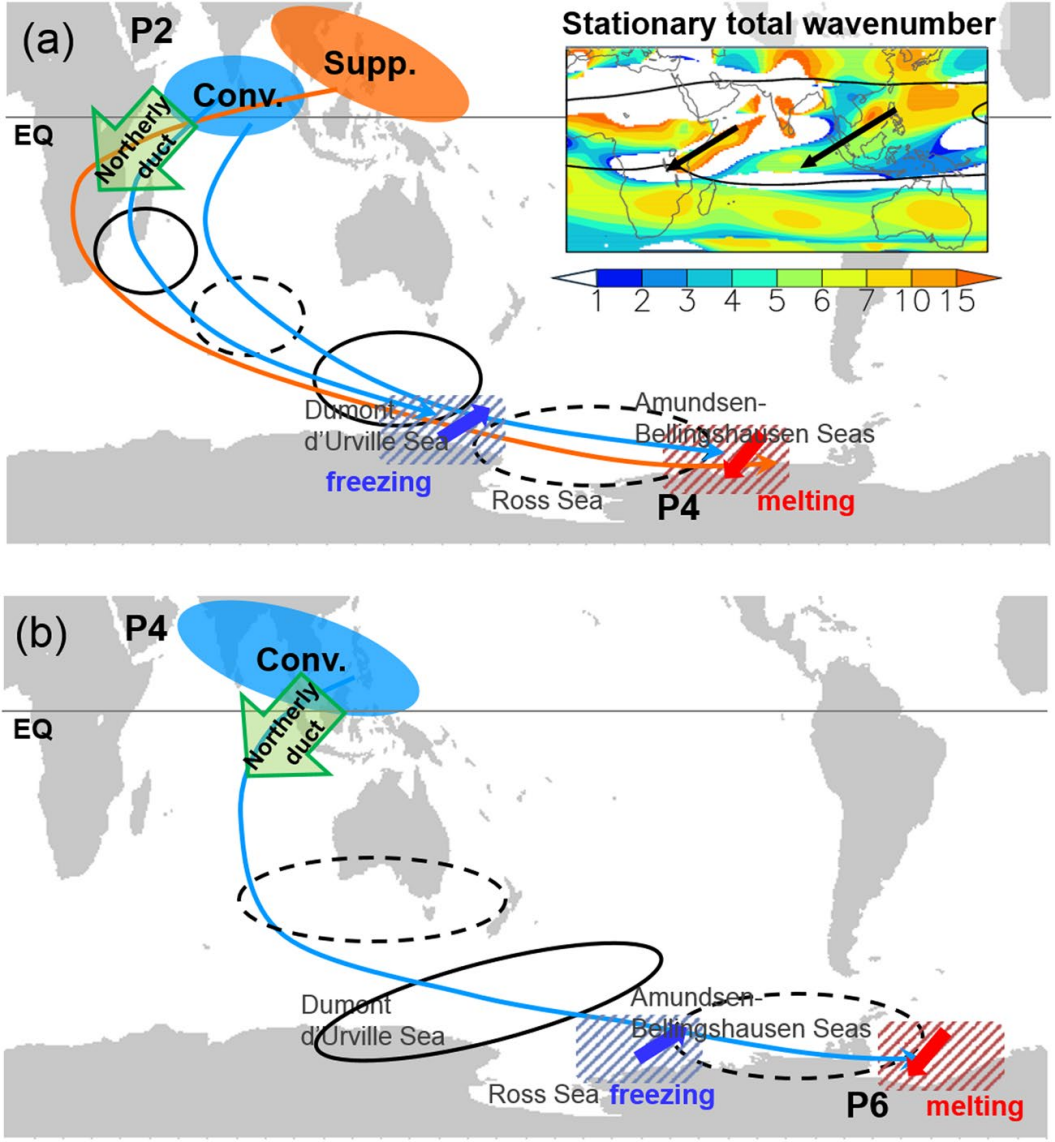
Dynamical mechanisms of MJO impact on the circulation and sea ice anomalies. Schematic illustrating the Rossby wave propagation from the MJO and formation mechanism of high-latitude circulation anomalies for the initial state of MJO (**a**) phase 2 and (**b**) phase 4. The black solid and dotted circles denote anticyclonic and cyclonic circulation anomalies, respectively. Green arrows near the equator depict the two northerly ducts. P4 in (**a**) and P6 in (**b**) denote the MJO phases at which the wave trains shown in the plots are established. The inset plot in (**a**) is the stationary total wavenumber (*K*_s_), with the two black arrows representing local peaks in this wavenumber. Red (blue) thick arrows denote poleward (equatorward) wind anomalies and red (blue) hatching indicates sea ice melting (freezing) area.

The results of the linear model simulations show that the high-latitude circulation anomaly propagates eastward as does the diabatic forcing (Supplementary Fig. S3). Subsequently, the wave train influences Antarctic sea ice variation as seen in Fig. 2b. Figure 4 summarizes the formation mechanism and the resulting change in sea ice extent. As shown in Fig. 3b,c,e, two dominant wave passages, referred to as the northerly ducts, are identified at the equator: the western Indian Ocean–central Africa and the Maritime Continent (green arrows in Fig. 4). These northerly ducts allow waves to propagate from the Northern Hemisphere into the Southern Hemisphere. This is possible since the dispersion relationship for the barotropic Rossby wave now includes the effect of the basic meridional flow and, thus, the waves can penetrate the tropical easterly region^38,39^. This is confirmed by calculating the stationary total wavenumber (*K*_s_), which implies the preferred region for wave propagation^40^. The inset plot in Fig. 4a shows the two wave passage routes.

### Prediction of probability of occurrence of the sea ice reduction by the MJO

**Figure 5:**
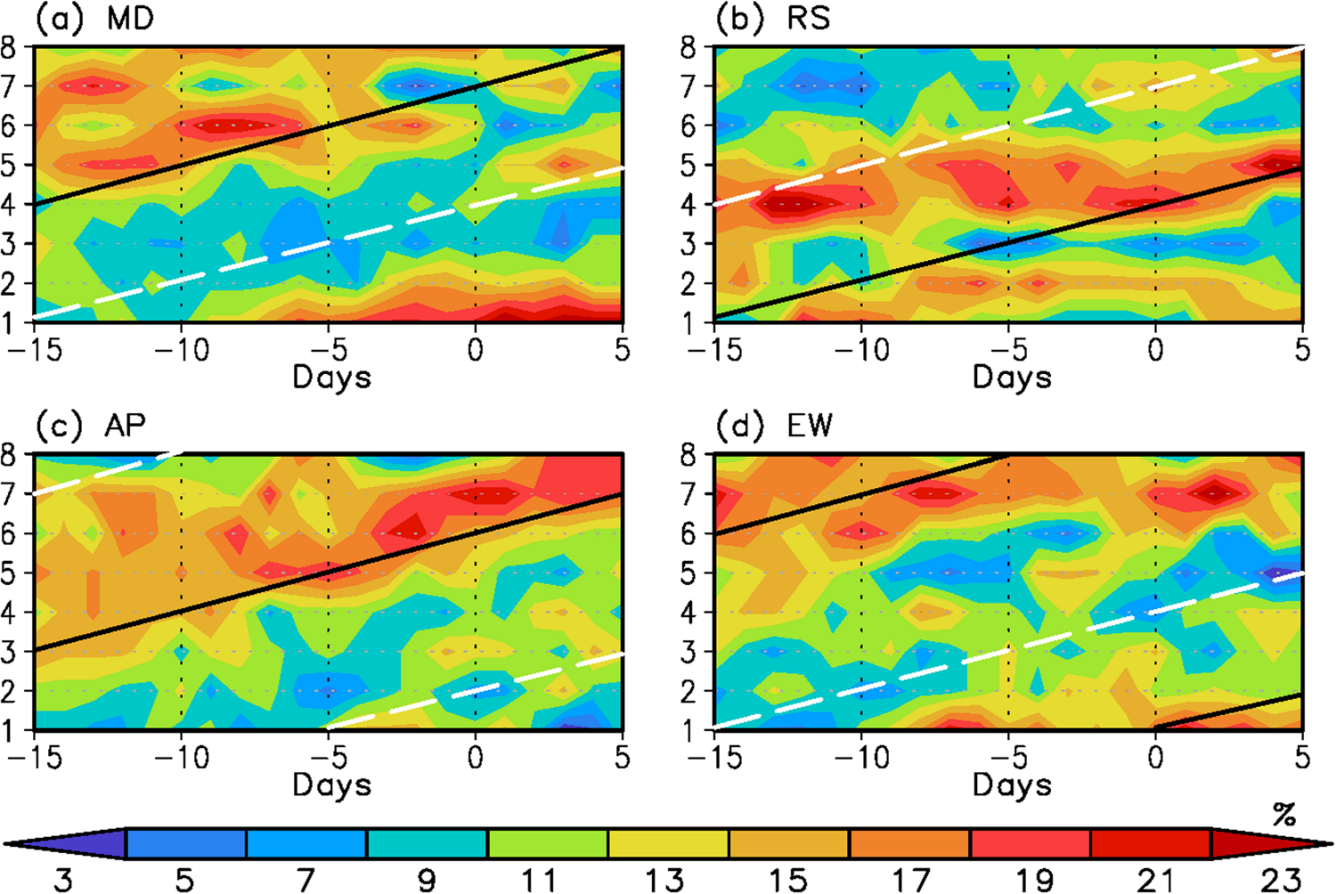
Prediction of probability of the sea ice extent decrease depending on MJO phases. Percentage of occurrence number of the MJO phases for four regions: (**a**) MD, (**b**) RS, (**c**) AP, and (**d**) EW as a function of lead or lag days relative to the date at which the total sea ice extent anomaly is smaller than −1.0 standard deviations and has a minimum value. The black solid (white dashed) line represents the approximated melting (freezing) dates estimated from day 0. Day 0 is determined from the peak melting (freezing) phase shown in the intraseasonally filtered sea ice extent anomaly (Fig. 2b). Then, the line is extended by considering that the time interval between adjacent MJO phases is 5 days.

It has been shown that on the intraseasonal time scale, the MJO induces the sea ice variation through Rossby wave propagation. Thus, a question arises as to whether the above physical process can be utilized in real prediction of the melting of Antarctic sea ice. For this, we use the total sea ice anomaly rather than the intraseasonally filtered anomaly. First, strong sea ice melting cases are selected based on events in which the sea ice extent anomaly is less than –1.0 standard deviations. Second, for lead and lag days, the percentage of occurrence number of the MJO phase is calculated for the four regions: the MD, RS, AP, and EW, as shown in Fig. 5, where lag day 0 is defined as the peak melting day.

The linearly approximated melting dates by the MJO effect are expressed as black solid lines for the lead and lag days (see the caption of Fig. 5 for the detailed definition of black solid line). The figure shows that over the MD, AP and EW, high percentage values tend to appear along the black line, indicating that ice melting over these regions can be predicted much earlier than at day 0 (Fig. 5a,c,d). Meanwhile, this kind of evolution pattern is not prominent in the case of the RS (Fig. 5b). It is likely that the sea ice over the RS is more affected by lower frequency forcings like those on the interannual time scales^41^, including the ENSO^42,43^. In addition, the figure shows that on the whole, for all regions, the MJO phase at which a high percentage is recorded at day 0 matches those MJO phases at which the peak intraseasonal sea ice reduction (see Fig. 2b) occurs.

## Summary and Discussion

This study is the first to reveal the MJO impact on Antarctic sea ice extent. During austral winter, the MJO convection anomaly exists over the equator and the Northern Hemisphere subtropics. Rossby waves emitted from the MJO propagate into the Southern Hemisphere through the two northerly ducts, which are located over the western Indian Ocean–central Africa and the Maritime Continent. The waves develop a teleconnection pattern, reaching the polar sea ice region. The enhanced and suppressed convection over the Indian Ocean and western North Pacific, respectively (e.g., MJO phase 2), produces a cyclonic circulation over the RS and Amundsen-Bellingshausen Seas at phases 3–4. The anomalous circulation is responsible for the sea ice extent anomaly, with an increase in the MD and a decrease in the RS. Meanwhile, the enhanced convection over the Indian subcontinent and Maritime Continent, representing MJO phase 4, induces an anticyclonic circulation anomaly over the MD and a cyclonic one over the Amundsen-Bellingshausen Seas and AP at phases 5–6, causing an increase in the sea ice extent in the RS and EW and a decrease in the AP. The physical processes are schematically summarized in Fig. 4.

The anomalous circulation generates the temperature advection by the meridional wind anomaly that modulates the sea ice variation. That is, the meridional wind anomaly leads to a temperature change within a day, while the latter tends to produce sea ice melting or freezing during the next 4 days. As the MJO propagates eastward from the Indian Ocean to the western North Pacific, the teleconnection also shifts to the east (which is due to a more zonally symmetric subtropical jet in the Southern Hemisphere compared to that of the Northern Hemisphere; this will be reported in other studies). Following the high-latitude circulation anomaly, the sea ice extent anomaly exhibits eastward-migrating behavior as well. It is suggested that the induced circulation pattern over the sea ice region near Antarctic is nearly antisymmetric for the opposite MJO convective forcing.

Intraseasonal variability of the sea ice extent accounts for up to 29% of the total variation in the sea ice extent. Although the amount seems to be rather small, this may have an implication on the real prediction of the sea ice extent using the MJO phase because the change in the total sea ice extent anomaly is also synchronous to the evolution of the MJO.

The eastward-migrating property of Antarctic sea ice concentration anomaly has been reported in earlier studies^33,36^, but the major causes of this movement have not been elucidated. This study shows that the variation in Antarctic sea ice extent is driven by the austral winter MJO.

## Methods

### Data

The 25 × 25 km gridded ice concentration data provided in the National Snow and Ice Data Center (NSIDC) are used. The data are derived by the brightness temperature field obtained from Nimbus-7 SMMR and DMSP SSM/I-SSMIS. Missing values in the data are replaced by linear temporal interpolation. Using the sea ice concentration, the sea ice extent is calculated by summing the areas of the grid cells where the sea ice concentration is greater than 15%. The local sea ice extent is reinterpolated into a regular 2.5° × 2.5° grid and meridionally summed over the Southern Hemisphere. The local sea ice extent is linearly detrended before analysis to exclude the global warming effect. The daily outgoing longwave radiation (OLR) data from the National Oceanic and Atmospheric Administration (NOAA) are utilized as a proxy for the tropical deep convection. For other atmospheric variables such as circulation and near surface temperature, the ERA-Interim reanalysis data provided by the European Centre for Medium-range Weather Forecasts (ECMWF) are used^44^. All obtained variables has 2.5° horizontal resolution. Intraseasonal variability for each variable is obtained by removing the annual cycle, which is calculated as the first three harmonics of the calendar mean cycle, and then by applying a 20–90-day Lanczos bandpass filter. Only austral winter, from June to August [JJA], is considered for the period of 1979 to 2015.

### Definition of the MJO index and composite

To categorize the evolution of the MJO, an empirical orthogonal function (EOF) analysis is performed with the filtered OLR anomaly in the tropics from 30°N to 30°S. The obtained first two EOF modes are used to divide the MJO propagation into 8 phases, according to the location of MJO convection^34^. The phase rotates from phase 1 to 8, and phases 1–4 represent an antisymmetric structure with respect to phases 5–8. The MJO index is calculated with the amplitude of the normalized first two PCs (i.e., $\sqrt{PC{1}^{2}+PC{2}^{2}})$. The obtained index is largely identical to the OLR-based MJO index (OMI) of Kiladis *et al*.^45^, implying insensitiveness to the choice of MJO index. Cases in which the MJO index is greater than 1.5 standard deviations are used for the composite analysis. To evaluate the statistical significance of the composite, a Monte Carlo test is conducted with 2000 samples. To obtain the null probability distribution of samples, the MJO index time series are randomly shifted to forward or backward but the variables to be tested are fixed.

### Experimental design

To investigate the circulation response to the MJO, a numerical experiment is performed using an atmospheric linear baroclinic model (LBM) with T42 horizontal resolution and 20 vertical levels in sigma coordinates. The LBM is based on the primitive equations and its code is available from http://ccsr.aori.u-tokyo.ac.jp/~lbm/sub/lbm.html. The horizontal diffusion is set such that the *e*-holding decay time of 6 h for the largest wavenumber, and the vertical diffusion is set with a damping time scale of 1000 days to suppress the vertical computational mode. The time scale of linear drag, which mimics Rayleigh friction and Newtonian damping, is set to 0.5 days for the lowest three levels, 1 day for the topmost two levels, and 20 days for the mid-levels. The experiment is initialized with the JJA-climatological background state, which contains the 3-dimensional wind, temperature, geopotential height, specific humidity, and mean sea level pressure. To imitate the MJO convection in the experiment, the areas where the OLR is greater (lesser) than 10 (−10) W m^−2^ in the composite map are chosen as diabatic cooling (heating). Since the MJO convection is not spatially stationary, the diabatic forcing is given for the first 7 days and then turned off. Note that the simulated circulation fields have weaker intensity than that observed due to the switching-off of the forcing. If the forcing is persistently applied, the intensity is similar to that of the observation, although the circulation pattern in the subtropics is somewhat different than that indicated from the observation.

### Ray tracing and stationary total wavenumber

Ray tracing analysis is used to demonstrate the evolution of the nondivergent barotropic Rossby wave activity^40,46^. The dispersion relationship, which includes the effect of the basic meridional flow on a beta plane, is written as^47^:

1 $$\omega ={\bar{u}}_{M}k+{\bar{v}}_{M}l+\frac{{\bar{q}}_{x}l-{\bar{q}}_{y}k}{{k}^{2}+{l}^{2}},$$

where *ω*, *k*, and *l* are the angular frequency and the zonal and meridional wavenumber, respectively, and ${\bar{u}}_{M}$ and ${\bar{v}}_{M}$ denote the basic zonal and meridional flow on a Mercator projection, respectively. The JJA-climatological 250-hPa horizontal wind is adopted as the basic flow. Variables ${\bar{q}}_{x}$ and ${\bar{q}}_{y}\,\,$are the zonal and meridional gradients of absolute vorticity, respectively, taking the forms ${\bar{q}}_{x}=\frac{1}{{a}^{2}\,\cos \,\phi }(\frac{{\partial }^{2}\bar{v}}{\partial {\lambda }^{2}}-\frac{{\partial }^{2}\bar{u}}{\partial \lambda \partial \phi }\,\cos \,\phi +\frac{\partial \bar{u}}{\partial \lambda }\,\sin \,\phi )$ and ${\bar{q}}_{y}={\bar{\beta }}_{M}+\frac{{\partial }^{2}\bar{v}}{\partial \lambda \partial \phi }+\,\tan \,\phi \frac{\partial \bar{v}}{\partial \lambda }$, where ${\bar{\beta }}_{M}=\partial f/\partial y-{\partial }^{2}{\bar{u}}_{M}/\partial {y}^{2}$ and *f* is the planetary vorticity. The case of *ω* = 0 produces the stationary Rossby wave. The zonal and meridional group velocities, calculated by the partial derivatives of equation (1) with respect to *k* and *l*, are expressed as:

2 $${c}_{gx}={\bar{u}}_{M}+\frac{({k}^{2}-{l}^{2}){\bar{q}}_{y}-2kl{\bar{q}}_{x}}{{K}^{4}}\,{\rm{and}}$$

3 $${c}_{gy}={\bar{v}}_{M}+\frac{({k}^{2}-{l}^{2}){\bar{q}}_{x}+2kl{\bar{q}}_{y}}{{K}^{4}}.$$

To calculate the group velocities, the cubic polynomial of *l*, which is derived from equation (1), should be solved. Afterwards, the ray path of the Rossby wave activity is determined by the following form: $\frac{dx}{dt}={c}_{gx}$ and $\frac{dy}{dt}={c}_{gy}$. The location of the ray is computed by using the fourth-order Runge-Kutta method^16,17,48^. Grids within the MJO convection are utilized as a starting point for seeding the Rossby wave, and the integration is carried out for 18 days. The zonal and meridional wavenumbers vary along the ray as follows:

4 $$\frac{{d}_{g}k}{dT}=-\,k\frac{\partial {\bar{u}}_{M}}{\partial X}-l\frac{\partial {\bar{v}}_{M}}{\partial X}-\frac{1}{{K}^{2}}(l\frac{\partial {\bar{q}}_{x}}{\partial X}-k\frac{\partial {\bar{q}}_{y}}{\partial X})\,{\rm{and}}$$

5 $$\frac{{d}_{g}l}{dT}=-\,k\frac{\partial {\bar{u}}_{M}}{\partial Y}-l\frac{\partial {\bar{v}}_{M}}{\partial Y}-\frac{1}{{K}^{2}}(l\frac{\partial {\bar{q}}_{x}}{\partial Y}-k\frac{\partial {\bar{q}}_{y}}{\partial Y}),$$

where ${d}_{g}/dT=\partial /\partial T+{u}_{g}\partial /\partial X+{v}_{g}\partial /\partial Y$ represents the Lagrangian derivative. The ray tracing technique helps to understand meridional propagation of the Rossby waves across a critical latitude, e.g., tropical easterly region.

The stationary total wavenumber (*K*_s_) in the Mercator coordinate system is written as ${{K}_{s}}^{2}=\frac{{\bar{q}}_{y}k-{\bar{q}}_{x}l}{{\bar{u}}_{M}k+{\bar{v}}_{M}l-\omega }$ . The larger *K*_s_value indicates the preferred region for wave propagation^40^.

## Supplementary information

Supplementary Figures

## Data Availability

All data are available to readers upon request.
