# Supplementary material for: Impact of the Madden-Julian oscillation on Antarctic sea ice and its dynamical mechanism
Source: Sci Rep. 2019 Jul 24;9:10761. doi: 10.1038/s41598-019-47150-3 (PMC6656727; doi:10.1038/s41598-019-47150-3)
Supplement: Supplementary file 1 — Supplementary Figures [file 41598_2019_47150_MOESM1_ESM.pdf]

Supplementary Information for:

Impact of the Madden-Julian oscillation on Antarctic sea ice and its dynamical mechanism

Authors: Hyun-Ju Lee[1] and Kyong-Hwan Seo[1,2]*

Affiliations:

[1]Department of Atmospheric Sciences, Division of Earth Environmental Systems, Pusan National University, Korea

[2]Research Center for Climate Sciences, Pusan National University, Korea

*Corresponding Author. E-mail: khseo@pusan.ac.kr

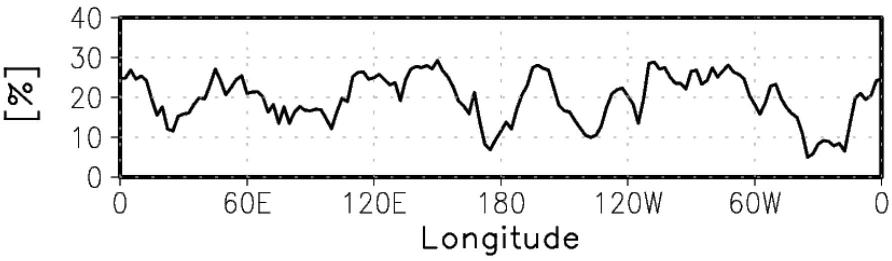

Supplementary Figure 1. Fraction of variation (%) of the sea ice extent explained by the intraseasonal variability over the Antarctic region. The fraction is calculated by the squared correlation coefficient between time series of total and filtered sea ice extent anomalies.

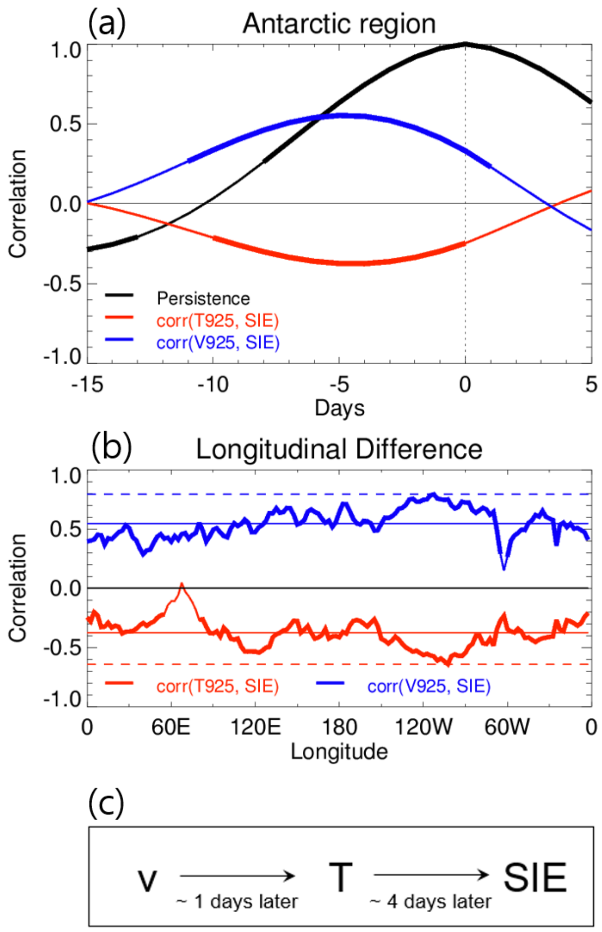

Supplementary Figure 2. Lead-lag relationship between the sea ice and atmospheric variables. (a) Lead-lag correlation coefficients between the sea ice extent anomaly and near-surface atmospheric variables—925-hPa air temperature (red solid), sea ice (black solid), and 925-hPa meridional wind (blue solid) anomalies over the Antarctic region. Here, the correlation coefficient is calculated using the variables that is meridionally averaged in same way as Fig. 2. (b) Correlation coefficients for the meridional wind and the temperature at 5-lead and 4-lead day times, respectively, on each zonal grid. Thick lines in (a) and (b) are statistically significant correlation coefficients at the 95% confidence level, based on the two-tailed Student *t*-test. (c) Schematic plot summarizing the relationships.

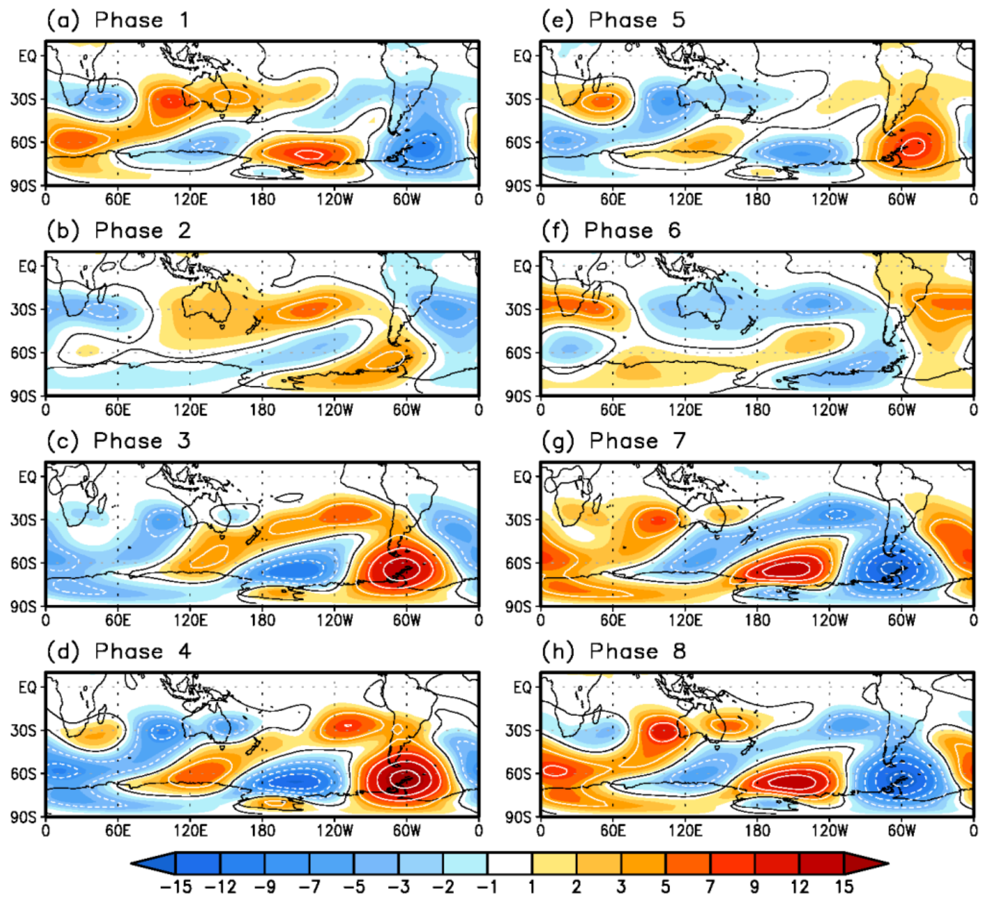

Supplementary Figure 3. Simulated circulation anomalies in response to the MJO. Same as Fig. 3 but for all eight MJO phases.
